# Pyroptosis in cardiovascular diseases: A bibliometric insight into current research and future directions

**DOI:** 10.1097/MD.0000000000050611

**Published:** 2026-09-11

**Authors:** Xiaohui Xie, Huatao Zhou, Jinfu Yang, Chengming Fan, Wenxiang Wang, Kele Qin

**Affiliations:** a Second Department of Thoracic Surgery, Hunan Cancer Hospital/The Affiliated Cancer Hospital of Xiangya School of Medicine, Central South University, Changsha, Hunan, China; b Medical School, Hunan University of Chinese Medicine, Changsha, Hunan, China; c Department of Cardiovascular Surgery, The Second Xiangya Hospital, Central South University, Changsha, Hunan, China.

**Keywords:** bibliometric analysis, cardiovascular diseases, CiteSpace, pyroptosis, VOSviewer

## Abstract

**Background:**

Pyroptosis, a unique form of programmed cell death, has emerged as an area of keen interest in cardiovascular disease (CVD) research. This study mapped the knowledge structure, research trends, and emerging directions of pyroptosis research in CVD.

**Methods:**

We performed a bibliometric analysis of English-language articles and reviews on pyroptosis in CVD published in the Web of Science Core Collection from January 1, 2006, to September 30, 2023. After independent eligibility assessment by 2 reviewers, the records were analyzed using VOSviewer, CiteSpace, and the bibliometrix R package. A targeted PubMed search conducted on July 30, 2026, was used only to contextualize representative developments published after the bibliometric cutoff.

**Results:**

We identified 3337 studies and reviews addressing pyroptosis in the context of CVD from 2006 to September 30, 2023. The analysis revealed that China had the highest number of publications, whereas the United States exhibited a more substantial impact in the field. Network analysis showed robust collaborative networks, particularly among the United States, China, and several European countries, with the top 10 contributing institutions hailing from the United States and China. Term frequency analysis identified “NLRP3 inflammasome,” “pyroptosis,” and “inflammation” as the most frequent keywords.

**Conclusion:**

This bibliometric analysis provides a reproducible historical baseline of cardiovascular pyroptosis research through September 2023. A targeted post-cutoff update indicates that subsequent work has increasingly focused on cell-specific gasdermin D and gasdermin E execution, immunometabolic and post-translational regulation, multimodal profiling, and direct gasdermin inhibition. These developments strengthen the trends identified in the original networks, while the continued predominance of preclinical evidence underscores the need for pathway-specific biomarkers and clinical validation.

## 1. Introduction

The term “pyroptosis” combines the Greek words “pyro” (meaning fire or fever) and “ptosis” (denoting falling) to describe an inflammatory form of programmed cell death.^[1]^ Initially identified in the 1990s, pyroptosis was observed when infections with Shigella flexneri or Salmonella in murine macrophages or human monocytes led to cell death, which was mistakenly classified as apoptosis.^[2]^ After 2001, distinctions between pyroptosis and apoptosis became clear, particularly the lytic and inflammatory nature of the former.^[1,3]^ The Nomenclature Committee on Cell Death officially defined pyroptosis in 2009 and 2012 as a form of cell death instigated by caspase-1 activation and later, in 2018, emphasized its regulation by the gasdermin (GSDM) protein family, which forms plasma membrane pores.^[4]^

It is currently believed that the activation and assembly of inflammasomes, through canonical pathways involving caspase-1 and noncanonical pathways triggered by caspase-4, -5, and -11, play a crucial role in pyroptosis.^[5]^ The canonical pyroptosis pathway relies on the caspase-1-mediated apoptotic route. Pattern recognition receptors (PRRs) recognize pathogen-associated molecular patterns or damage-associated molecular patterns, thus instigating pyroptosis.^[6]^ Caspase-1, a proenzyme or zymogen in its inactive state, becomes activated upon PRR engagement, triggering an inflammatory response.^[7]^ Stimulation of PRRs prompts the recruitment of caspase-1, either directly or mediated by ASC, constituting a caspase-1-dependent inflammasome.^[6,8]^ After inflammasome assembly, caspase-1 is activated, evolving from a zymogen to an active enzyme and initiating subsequent physiological responses.^[9]^ The noncanonical pathway entails cytoplasmic lipopolysaccharides directly provoking caspase-4/5/11 to orchestrate pyroptosis,^[10]^ with cleavage of the interleukin-1β (IL-1β) and interleukin-18 precursors solely dependent on caspase-1.^[11,12]^

The GSDM protein family, fundamental to pyroptosis, includes 6 paralogous genes: gasdermin A, gasdermin B, gasdermin C, gasdermin D (GSDMD), gasdermin E (GSDME; DFNA5), and DFNB59 (pejvakin).^[13,14]^ Since 2015, groundbreaking discoveries by Feng’s team have substantially advanced pyroptosis research, revealing GSDMD cleavage as a pivotal molecular mechanism in pyroptosis, with GSDMD acting as a direct effector molecule of inflammatory caspases.^[13,15]^ The proteolytic division of GSDMs liberates the N-terminal segment, adept at forming membrane apertures.^[16]^ These GSDM apertures disrupt cellular membrane integrity, leading to inflammatory cell demise and the extracellular discharge of cell contents, including inflammatory cytokines.^[17,18]^

Cardiovascular disease (CVD) remains a leading cause of mortality and diminished quality of life globally.^[19]^ Investigations have revealed a correlation between pyroptosis and the pathogenesis of numerous CVDs. Pyroptosis in endothelial cells,^[20]^ macrophages,^[21]^ and smooth muscle cells^[22]^ is intimately linked with the development and progression of atherosclerotic lesions and plaque stability.^[23]^ The presence of NOD-like receptor 3 (NLRP3)/IL-1β/caspase-1 expression has been documented in patients with diabetes, myocardial infarction, arrhythmia, and cardiac hypertrophy,^[24–26]^ and certain agents have demonstrated efficacy in ameliorating CVD symptoms by inhibiting pyroptosis.^[27]^

Bibliometric analysis quantitatively examines publication patterns and trends and provides complementary qualitative insights into a scientific field.^[28]^ It can characterize a field’s knowledge structure, collaborative networks, and evolution and compare contributions across authors, institutions, and journals.^[29]^ These methods can help identify research hotspots and emerging trends.^[30]^ However, bibliometric studies of pyroptosis in CVD remain scarce. Therefore, this study used bibliometric methods to quantitatively map the research landscape, knowledge structure, hotspots, and emerging directions of pyroptosis research in CVD.

## 2. Methods

### 2.1. Study design

This study was designed as a bibliometric analysis rather than a systematic review or meta-analysis. Its purpose was to quantitatively characterize publication output, collaboration networks, co-citation relationships, research hotspots, and emerging trends in the literature on pyroptosis and CVD. Because the study did not synthesize clinical effect estimates or assess the certainty of evidence from individual studies, meta-analytic procedures and study-level risk-of-bias assessment were not applicable.

### 2.2. Search strategy

We selected the Web of Science Core Collection (WOSCC) as the sole bibliographic source for this bibliometric analysis. WOSCC provides broad multidisciplinary coverage, standardized author, affiliation, keyword, cited-reference, and citation-link metadata, and full-record and cited-reference export formats that are directly compatible with CiteSpace, VOSviewer, and bibliometrix.^[30–35]^ These features are essential for reproducible co-authorship, co-citation, and keyword co-occurrence analyses. Although PubMed, Scopus, and Embase may identify additional records, their indexing structures, cited-reference fields, database coverage, and citation counts are not fully equivalent to those of WOSCC. Directly pooling records from these databases could introduce duplicate publications, inconsistent metadata, and noncomparable citation counts, which may alter network link strengths and rankings. Therefore, to maintain a single, internally consistent citation dataset and ensure reproducibility across all analyses, we retained WOSCC as the exclusive data source. The search covered publications from January 1, 2006, to September 30, 2023, and was performed using the following title-field query: (TI = “pyroptosis” OR TI = “pyroptotic” OR TI = “inflammasome” OR TI = “pyroptosome”) AND (TI = “cardiovascular*” OR TI = “cardiac*” OR TI = “heart*” OR TI = “hypertension*” OR TI = “cardio*” OR TI = “valve*” OR TI = “myocardial*” OR TI = “Pericardial*” OR TI = “arrhythmia*”). All records were retrieved and exported on October 22, 2023.

### 2.3. Eligibility criteria and literature screening

**Figure 1.**
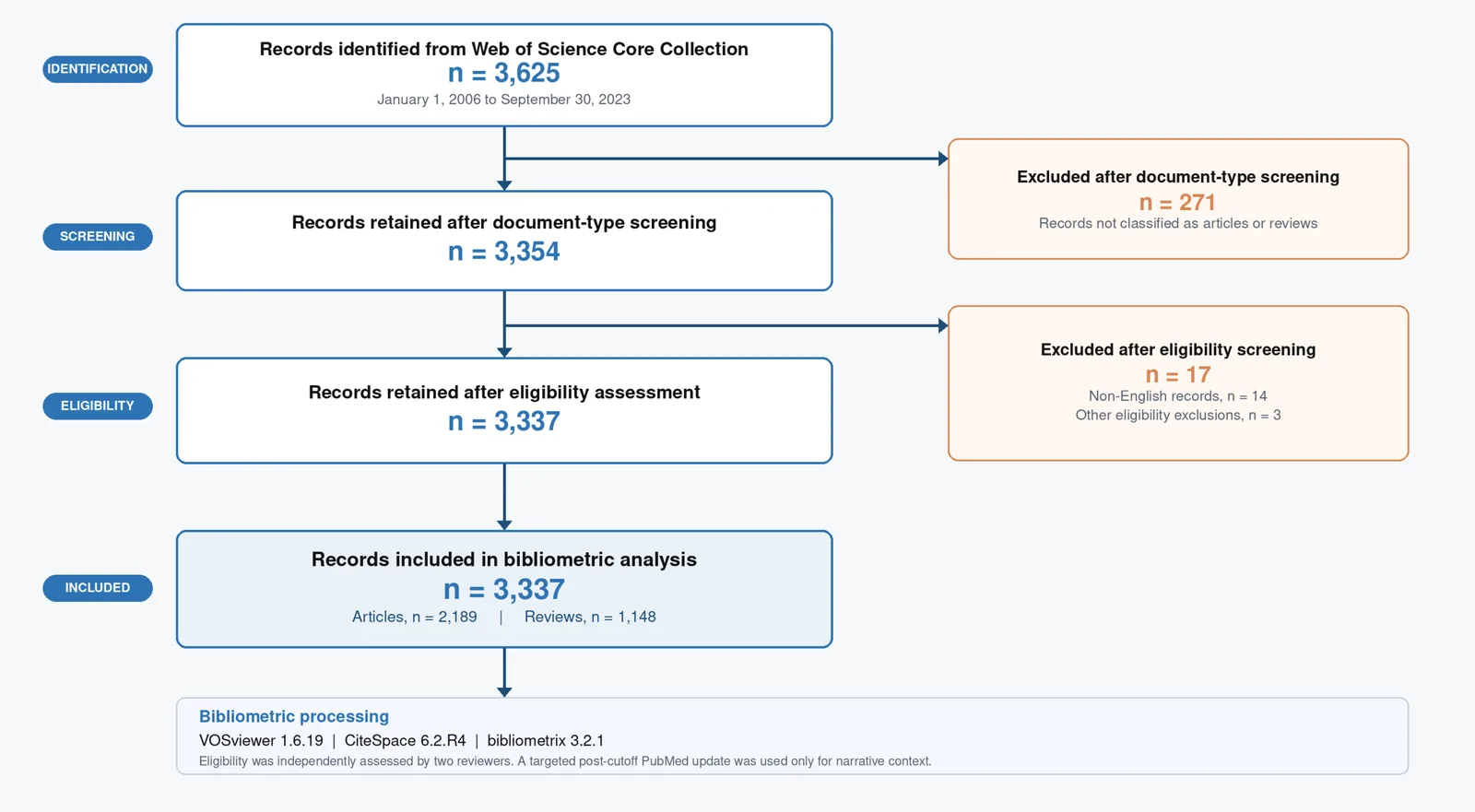
Process for literature screening.

Records were eligible if they were indexed in WOSCC; were published in English between January 1, 2006, and September 30, 2023; were classified by WOSCC as an Article or Review; and addressed pyroptosis- or inflammasome-related research in the context of CVD. We excluded non-English publications, document types other than articles and reviews (e.g., editorials, letters, meeting abstracts, corrections, and book chapters), publications outside the predefined period, exact duplicate records, and records that were not relevant to the topic after review of their titles and bibliographic information. Two reviewers independently conducted the search and eligibility assessment. Any disagreement was resolved through discussion or consultation with a third reviewer. The final dataset comprised 3337 records (2189 articles and 1148 reviews) and was exported in plain-text format with full records and cited references for bibliometric analysis. The identification and screening process is summarized in Figure 1.

### 2.4. Post-cutoff contextual literature update

**Figure 2.**
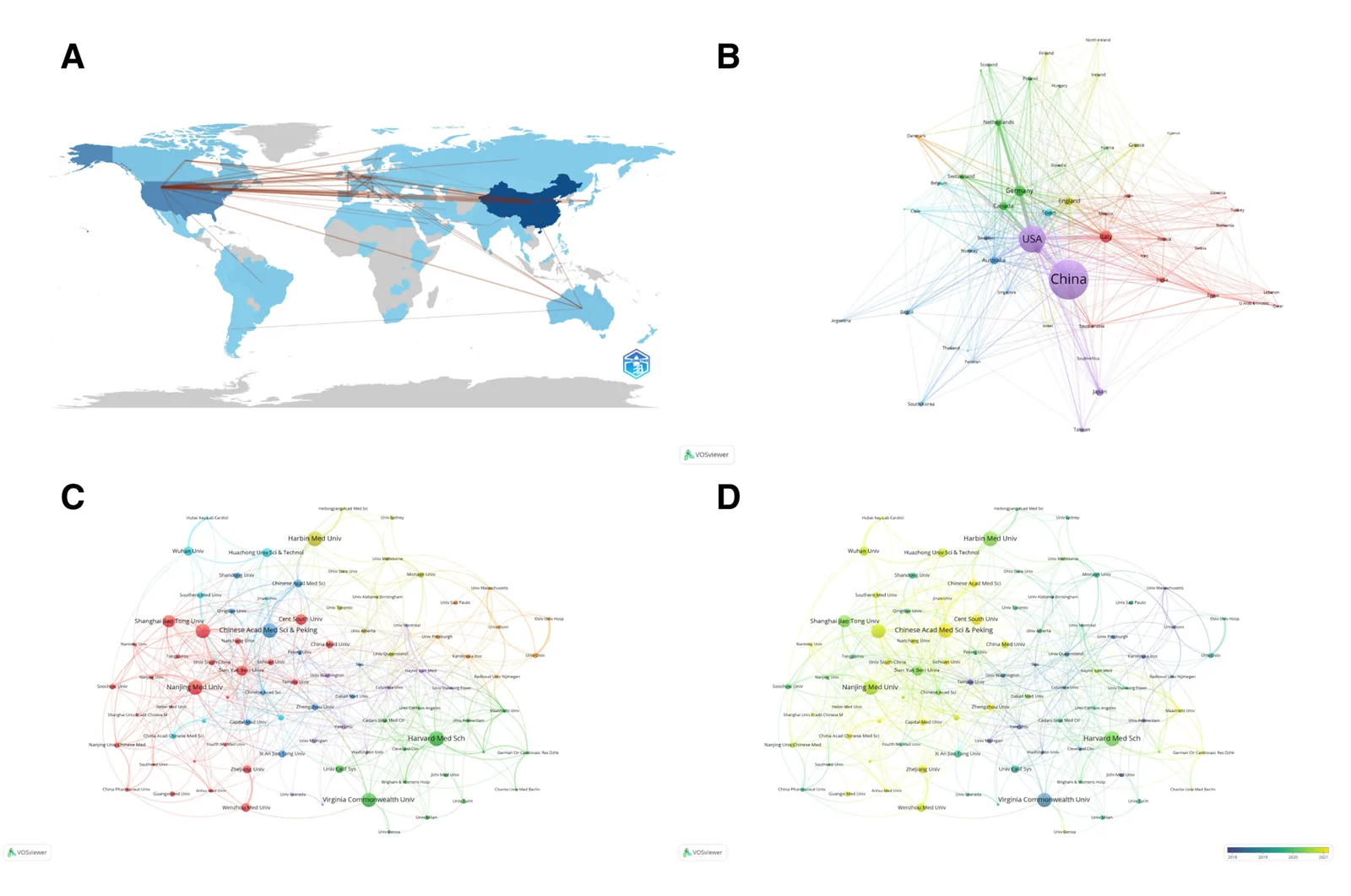
Geographical collaboration map (A), visualization of collaborating countries (B), research institutions (C), and analysis of their publication counts in recent years (D) related to pyroptosis in CVD. CVD = cardiovascular disease.

**Figure 3.**
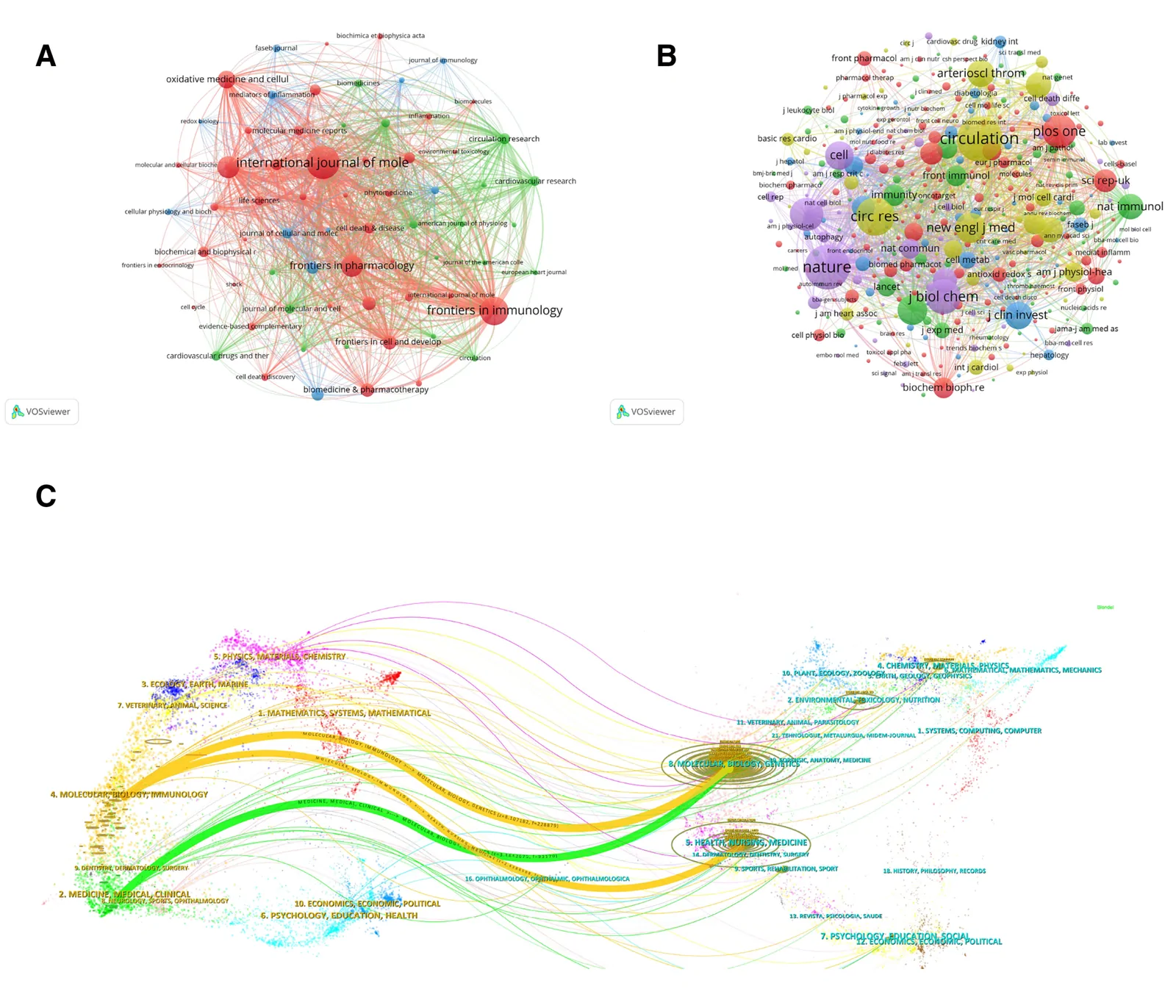
Visualization of contributing journals (A), co-cited journals (B), and the dual-map overlay of journals (C) in pyroptosis research within CVD. CVD = cardiovascular disease.

**Figure 4.**
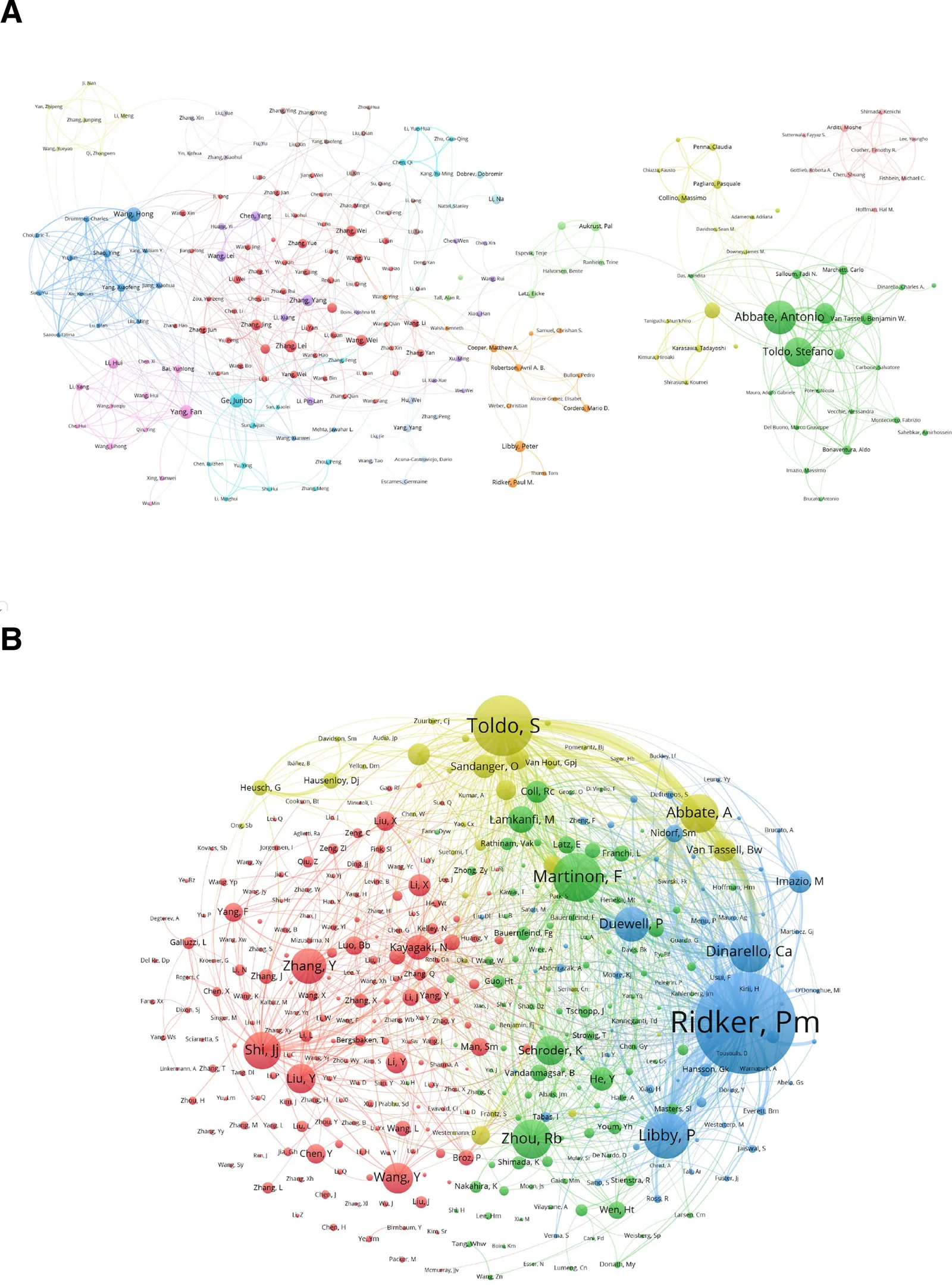
Visualization of authors (A) and co-cited authors (B) in research on pyroptosis in CVD. CVD = cardiovascular disease.

**Figure 5.**
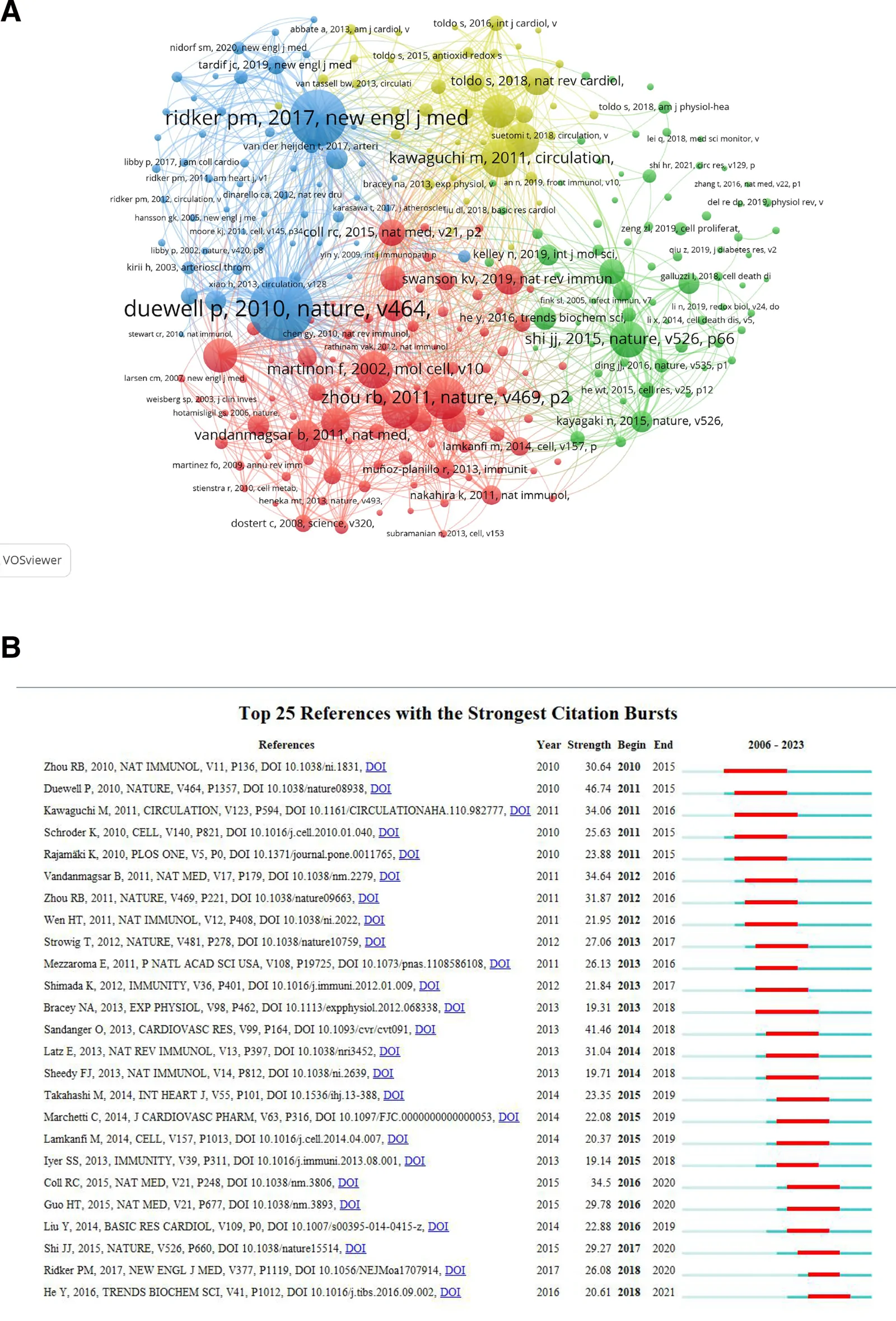
Visualization of co-cited references (A) and the top 25 references with strong citation bursts (B) in research on pyroptosis in CVD. CVD = cardiovascular disease.

**Figure 6.**
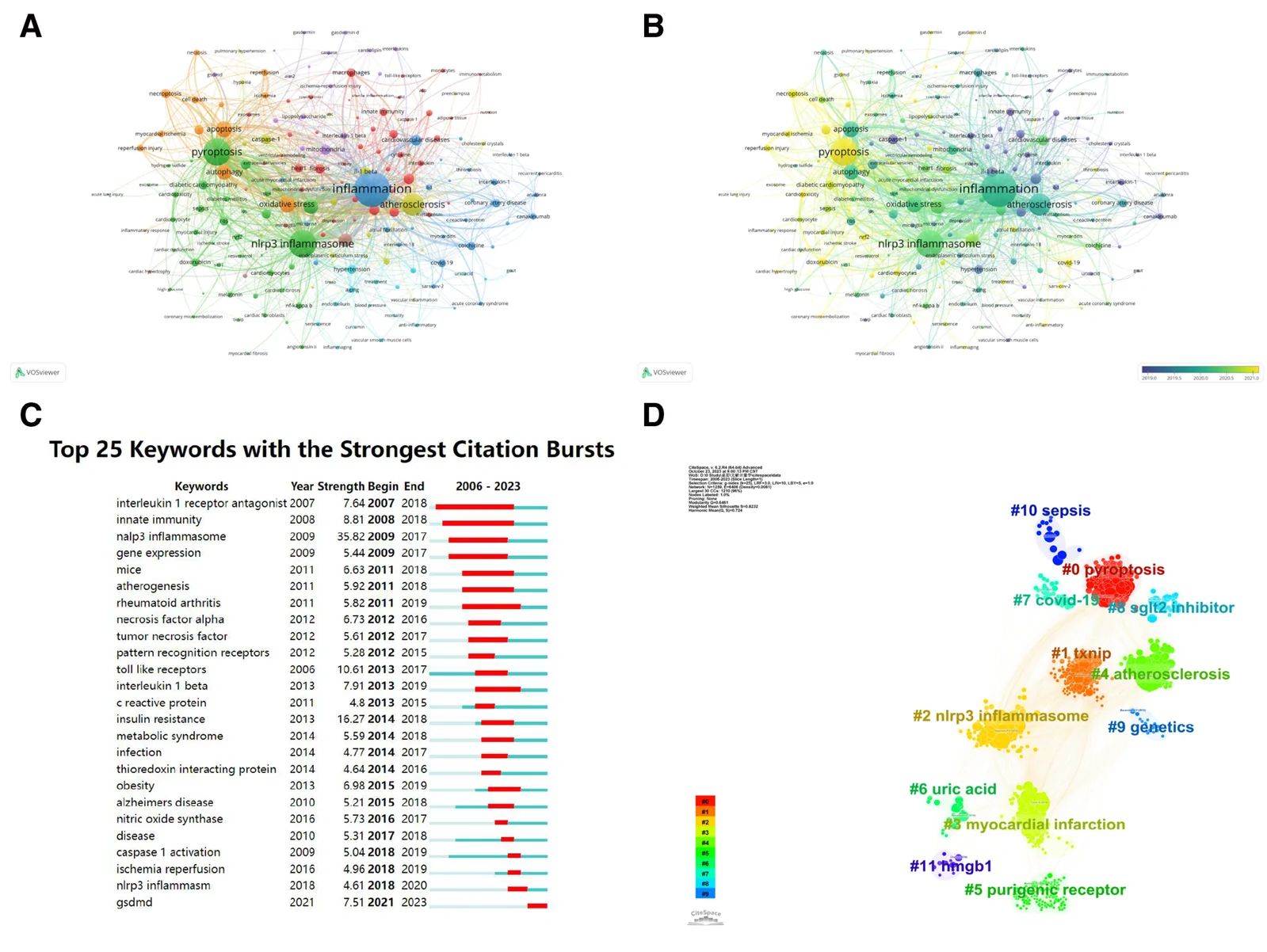
Visualization of keywords (A), temporal map of keywords (B), top 25 keywords with strong citation bursts (C), and keyword clusters (D) in research on pyroptosis in CVD. CVD = cardiovascular disease.

To address the rapidly evolving literature without changing the predefined bibliometric dataset, we conducted a targeted narrative search of PubMed on July 30, 2026. The search covered publications from October 1, 2023, to July 30, 2026, and combined the terms “pyroptosis,” “gasdermin,” or “inflammasome” with “cardiovascular,” “cardiac,” “myocardial,” “atherosclerosis,” “aneurysm,” or “pulmonary hypertension.” Representative original studies and major reviews were examined to identify developments that could clarify or extend the trends observed in the 2006 to 2023 networks. This contextual update was not a second bibliometric analysis or a systematic review. Post-cutoff publications were therefore not added to publication counts, citation metrics, clusters, burst analyses, or Figures 2 to 6.

### 2.5. Data analysis

VOSviewer (version 1.6.19; Centre for Science and Technology Studies [CWTS], Leiden University), a software program specifically designed for bibliometric analysis, is adept at deriving essential information from a plethora of publications.^[31]^ It is commonly used to construct collaboration, co-citation, and co-occurrence networks.^[32,36]^ In this research, VOSviewer was instrumental in conducting several analyses: examining countries and institutions, assessing journals and co-cited journals, evaluating authors and their co-cited counterparts, and analyzing keyword co-occurrence.

CiteSpace (version 6.2.R4), developed by Professor Chen C, is another bibliometric analysis and visualization instrument.^[33,34,37]^ Our study used CiteSpace to generate dual-map overlays of journals and to conduct analyses of references, keywords with citation bursts, and keyword clusters.

We utilized the R package “bibliometrix” (version 3.2.1; Massimo Aria & Corrado Cuccurullo; available at https://www.bibliometrix.org) to create a global publication distribution network on pyroptosis in CVD.^[35]^ The Journal Citation Reports 2022 provided journal impact factors and quartiles, while Microsoft Excel 2021 (Microsoft Corporation) was used for quantitative publication analysis.

## 3. Results

### 3.1. Publication trends in cardiovascular pyroptosis research

Introduced by American scientists in 2001, the study of pyroptosis has escalated, particularly since 2006, in the realm of CVD. Our research identified a total of 3337 related studies between 2006 and September 30, 2023, including 2189 articles and 1148 reviews. When examining annual publication growth, we discerned 2 distinct phases: phase I (2006–2017) and phase II (2018–2023). Phase I, as depicted in Figure S1, Supplemental Digital Content 1, was characterized by modest publication numbers, signifying the embryonic stage of pyroptosis research in CVD. The period from 2018 onwards witnessed a remarkable escalation in publications, reflecting significant strides in the field, heightened academic interest, and considerable scientific breakthroughs, thereby fostering enhanced exploration in this realm.

### 3.2. Geographical and institutional distribution

Publications originated from 94 countries and 3431 institutions. China produced the largest number of publications (1566, 45.19%), followed by the United States (837, 24.16%), Italy, Germany, and Canada (Table 1). Figure 2A and B show the geographical distribution and collaboration network of the 55 countries with at least 5 publications. Although China and the United States formed the most prominent bilateral collaboration, their network roles differed. China had substantially greater publication output, whereas its centrality was 0.07 compared with 0.39 for the United States. This contrast indicates that publication volume and network brokerage are not equivalent. The United States more frequently connected otherwise separate national groups, while China’s rapidly expanding contribution remained concentrated within fewer collaboration routes. The inclusion of Italy, Germany, and Canada among the 5 countries with the highest centrality further shows that European and North American nodes provide important bridges beyond the 2 largest producers.

**Table 1:** Top 10 countries and institutions by document count in pyroptosis research within CVD.

Rank	Country	Centrality	Count (%)	Institution	Centrality	Count (%)
1	China	0.07	1566 (34.21)	Harvard Medical School	0.07	78 (0.88)
2	USA	0.39	837 (18.28)	Harbin Medical University	0.01	76 (0.85)
3	Italy	0.14	215 (4.70)	Nanjing Medical University	0.03	75 (0.84)
4	Germany	0.12	189 (4.13)	Chinese Academy of Medical Sciences–Peking Union Medical College	0.02	74 (0.83)
5	Canada	0.08	126 (2.75)	Fudan University	0.04	73 (0.82)
6	Australia	0.11	116 (2.53)	Virginia Commonwealth University	0.03	73 (0.82)
7	England	0.11	115 (2.51)	Shanghai Jiao Tong University	0.05	63 (0.71)
8	Japan	0.04	112 (2.45)	University of California System	0.05	61 (0.68)
9	Spain	0.13	104 (2.27)	Sun Yat-sen University	0.04	56 (0.63)
10	Netherlands	0.03	85 (1.86)	Central South University	0.00	56 (0.63)

CVD = cardiovascular disease.

The institutional collaboration network in Figure 2C was dominated by Chinese and US institutions. Seven of the 10 most productive institutions were located in China and 3 in the United States, with Harvard Medical School (78 publications) and Harbin Medical University (76 publications) leading the ranking. In the temporal overlay in Figure 2D, yellow-toned nodes indicate more recent average publication activity, whereas purple-toned nodes represent institutions with an earlier average publication period. The Chinese Academy of Medical Sciences and Peking Union Medical College, Central South University, and Zhengzhou University therefore represent recently expanding contributors, while Virginia Commonwealth University, Yale University, and Temple University represent earlier-established contributors. This color contrast should not be interpreted as a direct decline in productivity. None of the leading institutions had a centrality above 0.1, indicating a decentralized network without a single coordinating hub. Such decentralization broadens participation, but it may also limit cross-center standardization and independent validation if collaboration remains concentrated within local institutional clusters.

### 3.3. Journals and co-cited journals

Cardiovascular pyroptosis studies were distributed across 883 journals (Fig. 3A). The *International Journal of Molecular Sciences* published the largest number of papers (114, 3.12%), followed by *Frontiers in Immunology* (88, 2.64%) and *Frontiers in Cardiovascular Medicine* (66, 1.98%). Among the 15 most productive journals, *Circulation Research* had the highest impact factor (20.1), followed by *Cell Death & Disease*^[9]^ (Table 2). The small share contributed by any single journal indicates that the research front is widely dispersed across molecular biology, immunology, cell death, and cardiovascular venues rather than concentrated within a single specialty.

**Table 2:** Top 15 journals with the highest publication output on pyroptosis in CVD.

Rank	Journal	Publications (%)	IF (JCR2022)	JCR quartile
1	*International Journal of Molecular Sciences*	104 (3.12)	6.2	Q1
2	*Frontiers in Immunology*	88 (2.64)	7.3	Q1
3	*Frontiers in Cardiovascular Medicine*	66 (1.98)	3.6	Q2
4	*Frontiers in Pharmacology*	65 (1.95)	5.6	Q1
5	*Oxidative Medicine and Cellular Longevity*	51 (1.53)	7.31	Q2
6	*Frontiers in Cell and Developmental Biology*	39 (1.17)	5.5	Q1
7	*International Immunopharmacology*	37 (1.11)	5.6	Q1
8	*Biomedicine & Pharmacotherapy*	37 (1.11)	7.5	Q1
9	*PLoS One*	33 (0.99)	3.7	Q2
10	*Scientific Reports*	33 (0.99)	4.6	Q2
11	*Cells*	31 (0.93)	6	Q2
12	*Circulation Research*	30 (0.90)	20.1	Q1
13	*Frontiers in Physiology*	30 (0.90)	4	Q2
14	*European Journal of Pharmacology*	30 (0.90)	5	Q1
15	*Cell Death & Disease*	29 (0.87)	9	Q1

CVD = cardiovascular disease, IF = impact factor, JCR = Journal Citation Reports.

The journal co-citation network in Figure 3B identified a more concentrated intellectual core. Among 8786 co-cited journals, 51 received more than 1000 co-citations. *Circulation* (6720 citations), *Nature* (6557), and *Circulation Research* (5300) occupied the leading positions, and the most highly co-cited journals were Q1 journals (Table 3). Importantly, the journals producing the largest number of current papers were not identical to those most frequently co-cited. This separation suggests a 2-level publication structure. Specialized and broad-scope journals disseminate much of the current output, whereas high-impact cardiovascular and fundamental science journals provide the principal knowledge base on which the field builds.

**Table 3:** Top 10 journals by co-citation frequency in pyroptosis research within CVD.

Rank	Cited journal	Citation	IF (JCR2022)	JCR quartile
1	*Circulation*	6720	37.8	Q1
2	*Nature*	6557	64.8	Q1
3	*Circulation Research*	5300	20.1	Q1
4	*Journal of Biological Chemistry*	4520	4.8	Q2
5	*PLoS One*	4248	3.7	Q2
6	*Proceedings of the National Academy of Sciences of the United States of America (PNAS*)	4239	11.1	Q1
7	*New England Journal of Medicine*	3691	158.5	Q1
8	*Journal of Immunology*	3586	4.4	Q2
9	*Cell*	3334	64.5	Q1
10	*The Journal of Clinical Investigation*	3164	15.9	Q1

CVD = cardiovascular disease, IF = impact factor, JCR = Journal Citation Reports.

The dual-map overlay in Figure 3C further clarifies the direction of knowledge transfer. The orange trajectories show that articles published in Molecular/Biology/Genetics and Health/Nursing/Medicine journals mainly cite research in Molecular/Biology/Immunology journals. The green trajectory shows that Medicine/Medical/Clinical journals also draw on Molecular/Biology/Genetics research. The convergence of both experimental and clinical citation paths on molecular, genetic, and immunological literature indicates that mechanistic science remains the common foundation of cardiovascular pyroptosis research. At the same time, the presence of a clinical citation pathway shows that these mechanisms are increasingly being incorporated into cardiovascular medicine, although stronger bidirectional links between clinical observations and experimental validation are still needed.

### 3.4. Authors and co-cited authors

A total of 17,922 authors contributed to this field between 2006 and 2023. Antonio Abbate was the most prolific author with 44 publications, followed by Stefano Toldo with 38 and Eleonora Mezzaroma with 27 (Table 4). Figure 4A shows that author productivity is organized into multiple color-coded collaboration communities rather than a single uniformly connected group. The concentration of output among repeatedly collaborating investigators suggests that sustained research programs have driven much of the field’s development, while the separation of collaboration clusters identifies an opportunity for greater exchange between teams. Figure 4B shows a different but complementary pattern. The most frequently co-cited authors were Paul M. Ridker (1301 citations), Stefano Toldo (782), Fabio Martinon (631), Peter Libby (596), and Charles A. Dinarello (514). These authors span clinical cardiovascular inflammation, atherosclerosis, innate immunity, and inflammasome biology. The difference between the productive-author and co-cited-author rankings distinguishes the current research front from its broader intellectual foundations, while Toldo’s presence in both rankings identifies a direct bridge between foundational work and ongoing cardiovascular pyroptosis research.

**Table 4:** Top 10 authors and co-cited authors in pyroptosis research within CVD.

Rank	Authors	Location	Count	Co-cited authors	Location	Citations
1	Antonio Abbate	USA	44	Paul M. Ridker	USA	1301
2	Stefano Toldo	USA	38	Stefano Toldo	USA	782
3	Eleonora Mezzaroma	USA	27	Fabio Martinon	Switzerland	631
4	Masafumi Takahashi	Japan	21	Peter Libby	USA	596
5	Hong Wang	China	19	Charles A Dinarello	USA	514
6	Fan Yang	China	18	Rongbin Zhou	Switzerland	504
7	Junbo Ge	China	18	Antonio Abbate	USA	498
8	Benjamin W. Van Tassell	USA	16	Jianjin Shi	China	493
9	Lei Zhang	China	16	Yang Zhang	China	456
10	Yang Zhang	China	15	Peter Duewell	Germany	450

CVD = cardiovascular disease.

### 3.5. Co-cited references and references with citation bursts

The dataset contained 164,976 co-cited references. Figure 5A visualizes how frequently cited papers form conceptual connections through shared co-citation. The most highly co-cited paper by Duewell et al, established a mechanistic link between cholesterol crystals, NLRP3 inflammasome activation, and atherogenesis (Table 5). Its prominence indicates that the danger-signal–inflammasome–atherosclerosis axis became a common conceptual bridge between basic inflammasome biology and CVD research. The network therefore identifies NLRP3 signaling not merely as a frequent topic, but as an organizing framework that connected previously separate areas of vascular biology, innate immunity, and inflammatory cell death.

**Table 5:** Top 10 most frequently co-cited references in pyroptosis research within CVD.

Rank	Reference	Citation	Year	First author	Journal
1	NLRP3 inflammasomes are required for atherogenesis and activated by cholesterol crystals	447	2010	Peter Duewell	*Nature*
2	Antiinflammatory therapy with canakinumab for atherosclerotic disease	398	2017	Paul M. Ridker	*New England Journal of Medicine*
3	A role for mitochondria in NLRP3 inflammasome activation	299	2011	Rongbin Zhou	*Nature*
4	Inflammasome activation of cardiac fibroblasts is essential for myocardial ischemia/reperfusion injury	262	2011	Masanori Kawaguchi	*Circulation*
5	The inflammasome: a molecular platform triggering activation of inflammatory caspases and processing of proIL-beta	258	2002	Fabio Martinon	*Molecular Cell*
6	The inflammasomes	258	2010	Kate Schroder	*Cell*
7	Cleavage of GSDMD by inflammatory caspases determines pyroptotic cell death	256	2015	Jianjin Shi	*Nature*
8	The inflammasome: a molecular platform triggering activation of inflammatory caspases and processing of proIL-beta	237	2002	Fabio Martinon	*Molecular Cell*
9	The NLRP3 inflammasome is up-regulated in cardiac fibroblasts and mediates myocardial ischaemia-reperfusion injury	236	2013	Øystein Sandanger	*Cardiovascular Research*
10	The inflammasome promotes adverse cardiac remodeling following acute myocardial infarction in the mouse	234	2011	Eleonora Mezzaroma	*PNAS*

CVD = cardiovascular disease, GSDMD = gasdermin D, NLRP3 = NOD-like receptor 3.

CiteSpace identified 372 references with citation bursts, and Figure 5B presents the 25 strongest bursts.^[38]^ The Duewell paper had the highest burst strength (46.74) from 2011 to 2015. Across the selected references, burst strengths ranged from 19.14 to 46.74 and generally lasted 2 to 5 years. The time-limited nature of these bursts indicates that the field developed through successive waves of rapidly adopted concepts rather than through a single stable research focus. When interpreted together with the recent emergence of GSDMD in the keyword analysis (Fig. 6B and C), the pattern suggests a transition from early emphasis on inflammasome activation and atherogenesis toward downstream GSDM-mediated execution and crosstalk among regulated cell-death pathways.

### 3.6. Hotspots and frontiers

Of 5556 author keywords from 3337 documents, 115 occurred in at least 15 documents and were included in the network analysis. In Figure 6A, inflammation, pyroptosis, the NLRP3 inflammasome, and atherosclerosis were among the most frequent terms. Their repeated co-occurrence with oxidative stress, apoptosis, and autophagy indicates that pyroptosis is increasingly studied as part of an interconnected inflammatory and regulated cell-death system rather than as an isolated pathway. The temporal overlay in Figure 6B supports this evolution. Atherosclerosis had an earlier average publication year (2019.90), whereas pyroptosis had a more recent average publication year (2021.91), and recent studies increasingly linked pyroptosis with necroptosis, apoptosis, autophagy, and ferroptosis. Figure 6C provides a complementary temporal signal. The NLRP3 inflammasome had the strongest keyword burst (35.82) from 2009 to 2017, reflecting the field’s foundational focus on inflammasome assembly, whereas the recent rise of GSDMD indicates a shift toward the pore-forming execution phase of pyroptosis. Figure 6D identified 12 keyword clusters with a modularity *Q* value of 0.65 and silhouette values above 0.7, indicating well-separated and internally coherent thematic communities. These clusters can be interpreted as 4 connected domains: molecular sensors and signaling pathways, cardiovascular and systemic disease contexts, metabolic and biochemical triggers, and genetic or environmental modifiers. Their relationships suggest a common research framework in which upstream stress signals converge on inflammasome activation and GSDM-mediated membrane injury, which then contributes to distinct cardiovascular phenotypes.

## 4. Discussion

This study utilized VOSviewer (version 1.6.19), CiteSpace (version 6.2.R4), and the R package “bibliometrix” (version 3.2.1) to scrutinize 3337 articles on pyroptosis in CVD, spanning from 2006 to September 30, 2023. Data from WOSCC were analyzed to ascertain spatial and temporal trends, influential authors, seminal articles, emergent hotspots, and the field’s frontiers.

### 4.1. Integrated interpretation of the network visualizations

Taken together, Figures 2 to 6 describe 3 connected levels of the field. Figure 2 defines the collaboration infrastructure, Figures 3 and 4 show how knowledge is disseminated and organized among journals and research teams, and Figures 5 and 6 reveal the intellectual foundations and thematic evolution of the literature. Read in combination, the maps show rapid growth accompanied by uneven international and institutional brokerage, a knowledge base anchored in molecular immunology and cardiovascular inflammation, and a conceptual transition from NLRP3-centered discovery toward GSDM-mediated execution and interactions among multiple regulated cell-death programs. Thus, the visualizations provide more than descriptive rankings. They identify structural and translational gaps that should guide future collaboration and study design.

### 4.2. General information

An uptick in annual publications, as depicted in Figure S1, Supplemental Digital Content 1, reflects the growing academic engagement with pyroptosis in CVD. From 2006 to 2017, a modest volume of literature suggests the field’s embryonic stage. Contrarily, from 2017 to 2023, there was an exponential growth in research, with 2022 witnessing a publication volume approximately 34 times higher than that in 2012. This surge in publications reflects the burgeoning interest and promising research trajectories in the study of pyroptosis within CVD.

Figure 2 reveals an important asymmetry between productivity and network connectivity. China and the United States contributed the largest shares of the literature, but the United States had much higher centrality. Centrality reflects a country’s ability to bridge otherwise separate collaboration groups and should not be interpreted as a direct measure of research quality. The pattern, therefore, suggests that China has developed substantial publication capacity, while the United States and several European countries play stronger brokerage roles across the global network. This distinction is important because continued growth in output alone may not yield broadly generalizable findings if datasets, methods, and validation studies remain concentrated within national or regional groups.

At the institutional level, the recent average publication years of several Chinese universities indicate the emergence of new high-output centers. However, the absence of an institution with centrality above 0.1 shows that no center currently coordinates knowledge exchange across the entire field. This decentralized structure can encourage diverse approaches, but it can also produce fragmented protocols, duplicated efforts, and limited external validation. Earlier-established centers in the United States and Europe and rapidly expanding centers in China therefore have complementary roles. Connecting them through multicenter consortia would combine mature collaboration networks with new patient populations and experimental capacity.

The practical implication of Figure 2 is that the principal collaboration gap is not insufficient publication activity but insufficient brokerage between rapidly growing institutional clusters. Future international networks should therefore prioritize shared definitions of pyroptosis, harmonized biospecimen and outcome protocols, interoperable datasets, and prospective independent replication across regions. Such arrangements would allow collaboration density to improve the reproducibility and clinical representativeness of the evidence rather than simply increasing publication counts.

Accordingly, the geographical and institutional maps support a specific conclusion: the field has achieved broad participation, but its next stage requires stronger connections among emerging and established centers so that network expansion is translated into standardized and externally validated research.

### 4.3. Status and quality of journals, authors, and studies

Figure 3A to C reveal a 2-level publication ecology. Current studies are widely disseminated through molecular, immunological, and cardiovascular journals, while the co-citation core is concentrated in high-impact journals such as *Circulation*, *Nature*, and *Circulation Research*. The dual-map overlay shows that both experimental and clinical publications rely heavily on molecular, genetic, and immunological evidence. This pattern confirms the interdisciplinary character of cardiovascular pyroptosis research, but it also shows that the field remains mechanistically anchored. A stronger translational literature will require clinical observations to generate testable mechanistic questions and mechanistic discoveries to be evaluated with clinically relevant endpoints.

#### 4.3.1. Interpretation of the author networks

Figure 4 separates the investigators driving current publication activity from the scholars who constitute the field’s intellectual base. The productive-author network is organized around stable collaborative teams, whereas the co-citation network integrates cardiovascular inflammation, atherosclerosis, cytokine biology, and inflammasome research. This difference explains why leading co-cited authors are not all specialists in pyroptosis. Their work supplied the concepts through which pyroptosis was connected to CVD. Greater collaboration across the color-coded author communities could help combine these complementary mechanistic and clinical perspectives.

As illustrated in Table 4 and Figure 4, Antonio Abbate is identified as the primary author in terms of articles published and is ranked seventh in terms of citation frequency. This underlines his considerable influence in the research of pyroptosis in CVD. Stationed at Virginia Commonwealth University, USA, Abbate contributed significantly through his 2018 publication “The NLRP3 inflammasome in acute myocardial infarction” in *Nature Reviews Cardiology*. This paper offers a thorough analysis of the NLRP3 inflammasome’s function in acute myocardial infarction, exploring its implications for inflammation-induced cell death, especially pyroptosis, in heart diseases. It emphasizes the therapeutic potential of targeting the NLRP3 inflammasome in the treatment of heart diseases and has received extensive citations. This work is pivotal in highlighting Abbate’s essential role in deciphering the intricate interactions between inflammation, cell death mechanisms, and cardiovascular health.

It’s important to note that Stefano Toldo, who collaborated on this critical work, is second in both the count of published articles and citation frequency, as Table 4 shows. The cooperative relationship between Antonio Abbate and Stefano Toldo is rooted in their shared research interests in cardiology, particularly focusing on the roles of inflammation and cell death mechanisms in CVDs. Their combined efforts have significantly propelled this field forward. Their partnership is characterized by concerted investigations into the molecular and mechanistic aspects of cell death pathways, with a focus on pyroptosis and its role in the onset of heart diseases. Together, they have probed the activation of the NLRP3 inflammasome, the function of pro-inflammatory cytokines, and potential therapeutic measures to regulate these pathways.^[39–41]^ The significance of their collaboration lies in its interdisciplinary nature, often blending clinical expertise with molecular and cellular biology approaches to bridge the divide between lab research and clinical applications. Their research not only enriches our comprehension of the complex interrelations between inflammation and cell death in CVDs but also lays the groundwork for novel treatment modalities. In essence, the collaboration between Stefano Toldo and Antonio Abbate acts as a propelling force in cardiology, offering a comprehensive and translational approach to tackle the complex issues associated with inflammation-related cell death in heart disease.

Renowned as the top co-cited author in Table 4, Paul M. Ridker is celebrated for his substantial impact on cardiovascular research, particularly regarding the role of inflammation in heart diseases. While pyroptosis and other cell death mechanisms have not been his main focus, Ridker’s studies have significantly broadened our comprehension of inflammation’s broader implications for cardiovascular wellness.

Among Ridker’s notable works, his 2017 paper titled “Antiinflammatory therapy with canakinumab for atherosclerotic disease,” holding the second position in Table 5’s co-cited references, stands out. This pivotal study, featured in the *New England Journal of Medicine*, delved into the effects of targeting inflammation – by inhibiting IL-1β – on cardiovascular event rates, independently of cholesterol levels. The study’s results highlighted Canakinumab’s role in diminishing high-sensitivity C-reactive protein levels, thereby confirming its effectiveness as an anti-inflammatory agent. This seminal research underscores the promise of IL-1β inhibition in potentially reducing the risk of cardiovascular events. It draws attention to the crucial role of inflammation in heart diseases, proposing an innovative approach to treatment that extends beyond merely lowering cholesterol. However, the research also brings attention to the potential risks linked to Canakinumab in clinical applications. Ridker’s work has been instrumental in steering further investigation into anti-inflammatory treatments for CVDs.^[42]^ Furthermore, Ridker has spearheaded clinical trials, such as the Justification for the Use of Statins in Prevention: an Intervention Trial Evaluating Rosuvastatin trial, to evaluate the efficacy of anti-inflammatory medications in diminishing cardiovascular risks. These trials, although not primarily focusing on cell death mechanisms, have illuminated how modulating inflammation can affect cardiovascular health.^[43]^ In essence, Paul M. Ridker’s contributions have indirectly enhanced our understanding of processes related to inflammation, including cell death mechanisms, highlighting the importance of addressing inflammation in the prevention and treatment of CVDs.

Peter Duewell, who occupies the tenth spot among co-cited authors in Table 4, is recognized for his highly influential work, “NLRP3 inflammasomes are required for atherogenesis and activated by cholesterol crystals.” Duewell’s research in the realm of pyroptosis has substantially progressed our grasp of the molecular intricacies and the pivotal importance of this inflammatory form of programmed cell death. His work has encompassed a range of areas, including demystifying the signaling pathways of pyroptosis, investigating its role in defending against infections, probing its impact on inflammatory conditions, and exploring potential therapeutic avenues targeting pathways related to pyroptosis.^[44–46]^ Duewell’s scholarly contributions have immensely deepened our understanding of pyroptosis, underscoring its significance in various physiological and pathological scenarios.

#### 4.3.2. Interpretation of co-citation and burst patterns

Figure 5 shows that the intellectual structure of the field was initially organized around NLRP3 activation, cholesterol-crystal sensing, and vascular inflammation. The finite duration of strong citation bursts indicates that individual concepts were rapidly adopted and then incorporated into the field’s common knowledge base. In combination with Figure 6, this pattern supports a maturation model in which research moved from identifying inflammasome triggers to defining downstream execution mechanisms and their interaction with other forms of regulated cell death.

### 4.4. Hotspots and frontiers

Figure 6 integrates co-occurrence, temporal overlay, citation-burst, and cluster analyses to show how the research focus has changed. The network is not composed of isolated themes. Instead, the 12 clusters form a connected sequence from metabolic, genetic, and damage-related triggers to inflammasome signaling, GSDM-mediated membrane injury, and CVD manifestations. The shift from an earlier NLRP3-centered burst to more recent GSDMD and cell-death crosstalk terms indicates increasing mechanistic specificity. For interpretive clarity, the clusters can be synthesized into the following 4 broader domains:

#### 4.4.1. Molecular mechanisms and pathways

Thioredoxin-interacting protein (TXNIP) and NLRP3 inflammasome: TXNIP and the NLRP3 inflammasome are key molecular components. TXNIP can activate the NLRP3 inflammasome, leading to the production of pro-inflammatory cytokines and pyroptosis.^[47]^ This pathway is a critical link between metabolic stress, inflammation, and cell death in CVDs.^[48]^

Purinergic receptors and HMGB1: Purinergic receptors such as P2X7 and molecules such as HMGB1 (high mobility group box 1) act as sensors or mediators linking cellular stress or damage to inflammatory responses. They play roles in activating pathways that lead to pyroptosis, illustrating how cellular distress signals contribute to CVD pathology.^[49–51]^

#### 4.4.2. Disease conditions and manifestations

Myocardial infarction and atherosclerosis: Pyroptosis plays a significant role in these cardiovascular conditions. In myocardial infarction, pyroptosis exacerbates tissue damage by contributing to cardiac cell death.^[52]^ In atherosclerosis, pyroptosis in vascular cells can lead to plaque formation and instability, triggering cardiovascular events.^[15]^

Sepsis: Though not a CVD, sepsis involves systemic inflammation and organ dysfunction, with mechanisms paralleling those in CVDs, including the role of pyroptosis.^[48]^

COVID-19: The pandemic highlighted the intersection between infectious diseases and cardiovascular health. Severe COVID-19 cases showed heightened inflammation and cardiac complications, prompting interest in the role of pyroptotic pathways in such interplays.^[53]^

#### 4.4.3. Biochemical factors and metabolic links

Uric acid: Elevated levels of uric acid have been associated with cardiovascular risk. Beyond its role in conditions like gout, uric acid has been implicated in activating inflammatory pathways, including those leading to pyroptosis, underscoring a biochemical facet of cardiovascular pathology.^[54,55]^

Sodium-glucose cotransporter 2 inhibitors: Initially used for diabetes management, these medications have shown cardiovascular benefits. Research into their potential anti-inflammatory effects, including modulation of pyroptotic pathways, reveals a therapeutic link between metabolic control and cardiovascular health.^[56,57]^

#### 4.4.4. Genetic and environmental influences

Genetics: Genetic research has revealed various mutations and polymorphisms that increase susceptibility to CVDs.^[58]^ Some of these genetic factors are linked to the regulation of inflammatory pathways, including pyroptosis.^[59]^ Understanding these genetic influences is crucial for identifying individuals at risk and for developing targeted therapies.^[60]^

Across these domains, the cross-cluster pattern suggests a shared pathogenic framework in which metabolic, genetic, or environmental stress activates inflammasome-related sensors, followed by GSDM-mediated membrane injury and amplification of inflammation in susceptible cardiovascular cell types. At the same time, the limited representation of validated clinical biomarkers and therapeutic trials in the dominant clusters highlights the distance between mechanistic prominence and clinical readiness. This network interpretation directly motivates the specific research gaps and actionable directions outlined below.

### 4.5. Emerging trends after the bibliometric cutoff (October 2023–July 2026)

The post-cutoff literature broadly supports the transition predicted by Figures 5 and 6 from upstream inflammasome activation toward GSDM execution, while adding greater cell-type and disease specificity. In atherosclerosis, genetic deletion of GSDMD reduced plaque burden, endothelial and macrophage death, inflammatory-cell infiltration, and neutrophil extracellular trap formation.^[61]^ A complementary study placed macrophage-derived GSDMD within a mitochondria–STING–IRF3/nuclear factor‑kappa B signaling circuit that supports inflammatory crosstalk in atherosclerosis.^[62]^ Beyond GSDMD, work in pulmonary hypertension linked cathepsin L-mediated bone morphogenetic protein receptor type 2 degradation to caspase-3/GSDME-dependent endothelial pyroptosis.^[63]^ Together, these studies indicate that the research front is moving from asking whether pyroptosis occurs in CVD to defining which GSDM acts in which cardiovascular cell type and through which disease-specific circuit.

A second emerging direction is the integration of metabolic regulation, post-translational modification, and human tissue-resolved analysis. A 2024 study reported that lactate dehydrogenase A enhanced lactylation of NLRP3 at K245 and increased its stability in experimental myocardial ischemia–reperfusion injury.^[64]^ In abdominal aortic aneurysm, single-cell RNA sequencing of human tissue, combined with experimental validation, implicated macrophage TNF receptor-associated factor 6 and the NLRP3/caspase-1/GSDMD pathway.^[65]^ These findings extend the earlier keyword clusters by showing how metabolic state and cell-specific regulatory nodes can determine pyroptotic activity, and they illustrate the field’s movement toward single-cell and multi-omics approaches linked to functional perturbation.

Therapeutic research has also become more target-specific. The small molecule GI-Y2 was reported to bind the Arg10 residue of GSDMD, reduce GSDMD-N membrane association, and attenuate atherosclerosis in mice. Macrophage membrane-coated nanoparticles were further used to improve delivery to atherosclerotic plaques.^[66]^ In parallel, an integrated mouse and human study linked aspirin-associated gut microorganisms and butyrate production to suppression of macrophage pyroptosis through the GPR109A–GSDMD pathway.^[67]^ These studies suggest that direct executioner inhibition, cell-targeted delivery, and microbiota-derived immunometabolic modulation may form the next therapeutic layer beyond broad inflammasome suppression.

These post-cutoff developments refine rather than overturn the original bibliometric conclusions. They reinforce the rise of GSDMD, broaden the execution phase to include GSDME, and add lactylation, single-cell analysis, targeted delivery, and the gut microbiota–metabolite interface as emerging themes. However, most of the new evidence remains preclinical, and pathway-specific clinical biomarkers and cardiovascular trials of direct GSDM inhibition remain limited. The recent literature therefore strengthens the mechanistic trajectory identified in Figures 5 and 6 while also confirming that clinical translation is the principal unresolved gap.

### 4.6. Research gaps and actionable future directions

The bibliometric patterns identified in this study indicate where attention has concentrated, but citation prominence alone does not establish biological causality, clinical validity, or therapeutic readiness. The principal gaps therefore concern not only which topics should be studied, but also which unanswered questions must be resolved and how they can be addressed with more rigorous and clinically relevant designs.

#### 4.6.1. Establish cell-type- and disease-stage-specific causality

A central unanswered question is whether pyroptosis is a causal driver of cardiovascular injury or a secondary consequence of inflammation and tissue damage, and which cell populations – cardiomyocytes, endothelial cells, vascular smooth muscle cells, fibroblasts, or infiltrating immune cells – are dominant at different disease stages. Much of the existing evidence is derived from acute cell-culture or rodent models, broad pharmacological inhibitors, and single time-point measurements. These designs may not distinguish pathway-specific effects from general anti-inflammatory effects or resolve temporal changes. Future studies should use inducible, cell-type-specific knockout or knock-in models targeting NLRP3, caspase-1/4/11, GSDMD, or GSDME, combined with lineage tracing, time-resolved sampling, loss-of-function and rescue experiments, and orthogonal confirmation in human cardiovascular tissue. Single-cell and spatial transcriptomic or proteomic analyses should be linked to functional perturbation rather than used only descriptively. Direct comparison with apoptosis, necroptosis, and ferroptosis is also required to determine whether observed injury is uniquely attributable to pyroptosis or reflects coordinated cell-death programs.

#### 4.6.2. Define and clinically validate pathway-specific biomarkers

No biomarker panel or diagnostic threshold currently identifies cardiovascular pyroptosis with adequate specificity. Frequently used measures such as IL-1β, interleukin-18, lactate dehydrogenase release, terminal deoxynucleotidyl transferase‑mediated dUTP‑biotin nick‑end labeling staining, or inflammasome-related messenger RNA can indicate inflammation or cell injury without demonstrating GSDM-mediated membrane permeabilization. Mechanistic studies should therefore apply a minimum evidentiary framework that combines upstream pathway activation (e.g., active inflammatory caspases), executioner cleavage (GSDMD or GSDME), membrane pore formation or loss of membrane integrity, and cytokine release. Clinical validation should proceed through prospective, multicenter cohorts with prespecified cardiovascular outcomes, repeated sampling, harmonized assays, and external validation. Where feasible, paired blood, tissue, and imaging measurements should be used to determine whether minimally invasive biomarkers reflect local cardiac or vascular pathway activity and add predictive value beyond established inflammatory and cardiovascular markers.

#### 4.6.3. Improve translational validity and therapeutic testing

It remains uncertain whether inhibiting pyroptosis provides benefit beyond broad suppression of inflammation, which molecular node offers the best therapeutic window, and when intervention should begin during acute injury or chronic disease. The predominance of short-term preclinical experiments, limited dose–response and pharmacokinetic/pharmacodynamic data, and insufficient assessment of infection risk restrict clinical translation. Preclinical studies should compare inhibitors of the inflammasome, inflammatory caspases, and GSDMs head-to-head, include target-engagement assays and pathway-specific rescue experiments, and test both early and delayed treatment. Models should incorporate aging, both sexes, major comorbidities, and concomitant standard-of-care therapies. Promising agents should then advance to biomarker-enriched early-phase trials with prespecified pharmacodynamic endpoints, cardiovascular efficacy signals, and systematic monitoring of infection, immune suppression, and off-target toxicity.

#### 4.6.4. Address disease heterogeneity and underrepresented populations

Current research is concentrated on atherosclerosis, myocardial infarction, and the NLRP3 inflammasome, leaving the contribution of pyroptosis to heart failure phenotypes, arrhythmias, valvular disease, and different cardiomyopathies less certain. The modifying effects of age, sex, ancestry, diabetes, obesity, chronic kidney disease, and cardiovascular medications also remain poorly resolved. Future work should use disease-specific models and adequately powered human cohorts with prespecified subgroup and interaction analyses. Human-induced pluripotent stem-cell-derived cardiomyocytes and vascular cells, organoids, and explanted tissue can complement animal models and help test whether mechanisms are conserved across patients. Recruitment and reporting should deliberately represent diverse populations so that candidate biomarkers and therapies are not validated only in narrow demographic or clinical groups.

#### 4.6.5. Strengthen methodological rigor, reproducibility, and data integration

The evidence base includes many small, single-center, cross-sectional, or exploratory studies with variable definitions of pyroptosis, limited blinding and randomization, and incomplete reporting of sample-size justification. Multi-omics associations are also often reported without causal or functional validation. Future experimental studies should use standardized nomenclature and outcome definitions, prespecified protocols, randomization, blinding, power calculations, appropriate biological replication, and independent confirmation. Animal studies should follow established reporting guidance, while clinical studies should preregister hypotheses and analysis plans. Open protocols, assay details, code, and deidentified data should be shared when possible, and negative findings should be reported to reduce publication bias. Integrative single-cell, spatial, genetic, and proteomic analyses should culminate in targeted perturbation and functional rescue to separate correlation from mechanism.

Collectively, these priorities support a staged translational framework: establish cell- and stage-specific causality; standardize pathway-specific measurements; validate biomarkers prospectively in representative human cohorts; and conduct biomarker-guided therapeutic studies with rigorous target-engagement and safety assessment. This sequence offers a concrete path from bibliometric signals to reproducible mechanisms and clinically testable interventions.

### 4.7. Limitations

This study has several limitations. First, the bibliometric dataset was derived exclusively from WOSCC. We selected WOSCC to obtain a single, standardized source of citation and cited-reference metadata and to avoid duplicate records and noncomparable citation counts arising from cross-database merging. Nevertheless, relevant publications indexed only in PubMed, Scopus, Embase, or other databases may have been omitted. The findings should therefore be interpreted as representing the WOSCC-indexed literature rather than the entire body of research on pyroptosis in CVD. Future studies should validate the present knowledge structure using harmonized and deduplicated datasets from multiple databases and should assess how database selection affects network patterns and rankings. Second, only English-language articles and reviews were included, which may have introduced language and document-type bias. Third, the quantitative analysis ends on September 30, 2023. Because publication output increased rapidly after 2017, post-cutoff studies may alter productive-author and institutional rankings, cluster weights, and the apparent prominence of burst keywords. Citation-based indicators also inherently disadvantage recent publications, which have had less time to accumulate citations and establish co-citation links. Fourth, the targeted update through July 30, 2026, was narrative and selective. It was intended to contextualize emerging developments and cannot substitute for rerunning the full bibliometric workflow with an updated, synchronized dataset. The network results should therefore be read as a historical baseline through September 2023 and updated periodically using time-sliced analyses. Finally, residual inconsistencies in author and institutional names and limitations inherent in VOSviewer, CiteSpace, and bibliometrix may have affected network construction and visualization.

## 5. Conclusion

This bibliometric analysis maps the WOSCC-indexed literature on pyroptosis in CVD through September 30, 2023. The networks show rapid growth, uneven international brokerage, and a conceptual progression from NLRP3-centered inflammasome discovery toward GSDM-mediated execution and crosstalk among regulated cell-death pathways. Representative literature published after the cutoff through July 2026 broadly validates this trajectory and extends it toward cell-specific GSDMD and GSDME mechanisms, lactylation and immunometabolic control, single-cell and multi-omics resolution, direct GSDM inhibition, targeted delivery, and microbiota-derived regulation. Most of these advances remain preclinical, making pathway-specific biomarker validation and rigorously designed translational studies the most urgent next steps.

Accordingly, the 2006 to 2023 network map should be regarded as a reproducible historical baseline rather than a fixed description of the current field. Periodic bibliometric updates, harmonized multi-database validation, stronger international collaboration, and prospective human studies will be essential to determine whether the rapidly expanding mechanistic literature can yield clinically useful biomarkers and therapies for CVD.

## Author contributions

**Data curation:** Huatao Zhou.

**Formal analysis:** Chengming Fan.

**Funding acquisition:** Wenxiang Wang.

**Investigation:** Xiaohui Xie.

**Methodology:** Xiaohui Xie, Jinfu Yang.

**Supervision:** Kele Qin, Jinfu Yang, Chengming Fan.

**Writing – original draft:** Huatao Zhou.

**Writing – review & editing:** Kele Qin.


